# Geschlechtsspezifische Unterschiede in der Adipositaschirurgie: Epidemiologie, Therapie und Ergebnisse

**DOI:** 10.1007/s00104-024-02149-z

**Published:** 2024-08-08

**Authors:** Patrick Téoule, Ema Pozek, Thomas Hielscher, Christoph Reißfelder, Christine Stier, Mirko Otto, Sebastian Schölch

**Affiliations:** 1 Chirurgische Klinik, Universitätsmedizin Mannheim, Medizinische Fakultät Mannheim, Ruprecht-Karls-Universität Heidelberg, Theodor-Kutzer-Ufer 1–3, 68167 Heidelberg, Deutschland; 2 Abteilung für Biostatistik (C060), Deutsches Krebsforschungszentrum, Heidelberg, Deutschland; 3 NW-KKE Translationale Chirurgische Onkologie (A430), Deutsches Krebsforschungszentrum, Im Neuenheimer Feld 280, 69120 Heidelberg, Deutschland; 4 DKFZ Hector Cancer Institute, University Medical Center Mannheim, Mannheim, Deutschland

**Keywords:** Bariatrische Chirurgie, Komorbiditäten, Männer, Frauen, Gewichtsverlust, Bariatric surgery, Comorbidities, Men, Women, Weight loss

## Abstract

In dieser Studie werden geschlechtsspezifische Unterschiede bei Adipositas und deren Behandlung mittels metabolisch/bariatrischer Chirurgie (MBS) untersucht. Die Datenbasis umfasst 2393 Patient*innen (1725 Frauen, 668 Männer) aus einem High-volume-Zentrum für bariatrische Chirurgie. Es wurden demographische, perioperative und Nachuntersuchungsdaten retrospektiv analysiert. Männer wiesen einen signifikant höheren Body-Mass-Index (BMI) und häufiger adipositasassoziierte Erkrankungen auf. Frauen stellten trotz der höheren Prävalenz der Adipositas bei Männern 80 % der operierten Patient*innen. Männer hatten im Mittel längere Operationszeiten bei gleichen Komplikationsraten. Postoperativ zeigte sich bei beiden Geschlechtern ein signifikanter Rückgang des Übergewichts, der bei Frauen etwas stärker ausgeprägt war. Die Studie unterstreicht insbesondere die Notwendigkeit, Männer besser zur Adipositasbehandlung zu motivieren, um gesundheitliche Folgen in dieser Bevölkerungsgruppe zu reduzieren.

**Zusatzmaterial online:**

Zusätzliche Informationen sind in der Online-Version dieses Artikels (10.1007/s00104-024-02149-z) enthalten.

## Abstract (en)

This study investigates gender-specific differences in obesity and the treatment by metabolic bariatric surgery (MBS). The database includes 2393 patients (1725 women, 668 men) from for a high-volume center for bariatric surgery. Demographic, perioperative and follow-up data were retrospectively analyzed. Men had a significantly higher body mass index (BMI) and more frequent obesity-associated diseases. Despite the higher prevalence of obesity in men, women accounted for 80% of the surgical patients. On average men had longer operation times but with the same complication rates. Postoperatively, both sexes experienced a significant reduction in excess body weight, which was slightly more pronounced in women. The study particularly emphasizes the need to better motivate men to undergo obesity treatment in order to reduce the health consequences of morbid obesity in this population group.

## Hintergrund

Weltweit hat sich die Prävalenz der Adipositas seit 1975 fast verdreifacht und stellt ein immer größeres gesundheitspolitisches Problem dar [20]. Die Prävalenz nimmt weiterhin kontinuierlich zu und in der Folge betreffen Übergewicht und Adipositas inzwischen mehr als 50 % der Bevölkerung in Europa [18]. Die Erkrankungsdauer und der Grad der Adipositas sind mit einer steigenden Inzidenz an adipositasassoziierten Erkrankungen vergesellschaftet. Diese Komorbiditäten beeinträchtigen sowohl die Lebenserwartung als auch die Lebensqualität der Betroffenen erheblich [5].

### Geschlechtsspezifische Unterschiede bei der Adipositas

Körperwahrnehmung und Selbstbild werden stark von gesellschaftlichen Normen beeinflusst, was beim Vorliegen einer Adipositas zu sozialer Stigmatisierung führen kann [8]. Dies ist ein geschlechtsspezifisches Problem, da Übergewicht beim weiblichen Geschlecht gesellschaftlich stärker stigmatisiert erscheint als bei Männern. So ist bei Männern die abdominelle Adipositas bis zu einem gewissen Grad gesellschaftlich akzeptiert. Zudem spielen psychosoziale Faktoren wie Leidensdruck, Stressbewältigungsfähigkeiten und soziale Unterstützung eine Rolle bei der Inanspruchnahme und dem Erfolg von Therapien. Dies kann zu unterschiedlicher Therapiemotivation und Compliance zwischen den Geschlechtern führen.

Fast 80 % der Patient*innen, die sich für eine Operation entscheiden, sind Frauen

Darüber hinaus gibt es biologische und anatomische Unterschiede zwischen den Geschlechtern. Während Frauen mehr subkutanes Fettgewebe besitzen, spielt bei Männern vor allem viszerales Fettgewebe eine Rolle [17, 19]. Beide Fettverteilungsmuster beeinflussen sowohl die Eigen- als auch die Fremdkörperwahrnehmung. Die gesundheitlich negativeren Auswirkungen hat viszerales Fettgewebe, was zu einer höheren Inzidenz adipositasassoziierter metabolischer Erkrankungen bei Männern führt [9]. Darüber hinaus gibt es weitere Unterschiede im Stoffwechsel zwischen Männern und Frauen, wie z. B. einen gewichtsunabhängig geringeren Grundumsatz von Frauen [2], was sich wiederum auf das Therapieansprechen auswirken kann. Zusätzlich bestehen geschlechtsspezifische hormonelle Unterschiede, die das Therapieansprechen ebenfalls beeinflussen können [11].

Die derzeit effektivste Behandlungsmethode der Adipositas ist die metabolische/bariatrische Chirurgie (MBS). Diese wird von den Geschlechtern unterschiedlich stark in Anspruch genommen – weltweit sind fast 80 % der Patient*innen, die sich für eine Operation entscheiden, Frauen [10], obwohl die Verteilung der Adipositas auf beide Geschlechter weitgehend gleich ist. In Bezug auf Begleiterkrankungen ist Adipositas bei Männern häufiger mit obstruktiver Schlafapnoe, arterieller Hypertonie, Diabetes und Dyslipidämie zum Zeitpunkt der Operation assoziiert [17]. Im kürzlich veröffentlichten *Global Registry Report 2023* der International Federation of the Surgery of Obesity and Metabolic Disorders (IFSO; [10]) wurden weltweit knapp 500.000 Eingriffe aus den Mitgliedsländern zusammengetragen und ausgewertet. Aus diesen Daten lassen sich internationale und länderspezifische geschlechtsspezifische Unterschiede ableiten. Daten aus Deutschland fehlen. Daher sollen im Rahmen dieser Arbeit geschlechtsspezifische Untersuchungen der Adipositasepidemie und ihrer Therapie bezogen auf patient*innenspezifische und perioperative Faktoren aus einem universitären Exzellenzentrum für MBS dargestellt werden.

## Methoden

### Datenbank

Diese Arbeit wurde mithilfe des Registers StuDoQ|Metabolische & Bariatrische Erkrankungen des Studien‑, Dokumentations- und Qualitätszentrums (StuDoQ) der Deutschen Gesellschaft für Allgemein- und Viszeralchirurgie (DGAV) durchgeführt. Es wurden nur Datensätze aus dem Exzellenzzentrum (DGAV) für Bariatrische und Metabolische Chirurgie der Universitätsmedizin Mannheim verwendet und retrospektiv ausgewertet.

Eingeschlossen wurden Patient*innen, die sich zwischen dem 01.10.2015 und dem 31.12.2023 erstmalig in unserem Zentrum zur Beratung vorgestellt haben. Ein positives Ethikvotum der Ethikkommission II der Universität Heidelberg liegt unter dem Aktenzeichen 2012-215N-MA vor. Die Studie wurde im Einklang mit der Deklaration von Helsinki (in der aktuellsten Revision vom Oktober 2013) durchgeführt.

Für die Analysen wurden zwei verschiedene Populationen betrachtet. Die demographischen Daten basieren auf allen Patient*innen aus dem Register, für die bei der Erstuntersuchung gültige Daten zum Körpergewicht vorlagen. Die Analyse der perioperativen und Nachuntersuchungsdaten basiert auf den Patient*innen, die bei der Erstuntersuchung gültige Gewichtsmessungen hatten, operiert wurden und für die die durchgeführte Operationsmethode bekannt ist.

Es wurden insgesamt 5 Visitenzeitpunkte untersucht: Erstkontakt (üblicherweise 6–7 Monate präoperativ, gefolgt von einem 6‑monatigen konservativen Therapieversuch), perioperativ, Follow-up (FU) 1 (3 Monate postoperativ), FU2 (12 Monate postoperativ), FU3 (24 Monate postoperativ). Für spätere Zeitpunkte war aufgrund der geringen Patient*innenadhärenz und damit fehlender Daten eine Auswertung nicht möglich (vgl. Tabelle S1 im Onlinezusatzmaterial).

### Statistische Analyse

Der prozentuale Verlust an Übergewicht („excess weight loss“, EWL) zu einem bestimmten Nachsorgezeitpunkt ist definiert als ([PräOP Gewicht − FU Gewicht] / [PräOP Gewicht − Idealgewicht] * 100), mit Idealgewicht entsprechend einem Body-Mass-Index (BMI) von 25 kg/m^2^ [3].

Die Änderung im BMI zur präoperativen Messung bzw. des prozentualen Rückgangs an Übergewicht wurde für jeden Nachsorgezeitpunkt auf Unterschiede zwischen Männern und Frauen mit einem longitudinalen ANCOVA-Modell getestet. Dazu wurde ein gemischtes lineares Modell mit zufälligem Patient*inneneffekt und den festen Einflussfaktoren Zeitpunkt und Geschlecht sowie deren Interaktion angepasst, zusätzlich adjustiert für den präoperativen BMI und das Patient*innenalter.

Ein Zweistichproben-Wilcoxon*-*Test wurde durchgeführt, um kontinuierliche Variablen in den Geschlechtsgruppen zu vergleichen. Zum Vergleich kategorialer Variablen in den Geschlechtsgruppen wurde der exakte Fisher*-*Test verwendet.

Perioperative Daten wurden zwischen den Gruppen getrennt für die beiden Operationsmethoden verglichen. Der Wilcoxon-Test wurde verwendet, um die Geschlechter hinsichtlich der Operationsdauer zu vergleichen. Alle anderen in den Tabellen aufgeführten Variablen sind kategorial und die in den Tabellen dargestellten *p*-Werte sind Ergebnisse des exakten Fisher-Tests. Aufgrund des explorativen Charakters dieser Registerstudie wurden die *p*-Werte nicht für multiples Testen angepasst. Die Varianz wurde jeweils in Klammern als ± Standardabweichung („standard deviation“, SD) angegeben. Ein *p*-Wert von *p* < 0,05 wurde als statistisch signifikant betrachtet.

## Ergebnisse

### Demographische Daten der Studienpopulation

In Tab. 1 sind die demographischen Daten zusammengefasst.

**Tab. 1:** Demographische Daten der Patient*innen bei Erstvorstellung

	Frauen (*n* = 1725)	Männer (*n* = 668)	Gesamt (*n* = 2393)	*p*-Wert
*Alter (Jahre)*				**0,007**
Mittelwert (SD)	41,3 (11,8)	42,8 (12,0)	41,7 (11,9)	
Median [Min, Max]	40,7 [15,5, 76,0]	42,3 [16,1, 74,7]	41,0 [15,5, 76,0]	
*Körpergröße (cm)*				**<0,001**
Mittelwert (SD)	166 (6,89)	180 (7,35)	170 (9,59)	
Median [Min, Max]	165 [106, 194]	180 [140, 201]	169 [106, 201]	
*Gewicht bei Erstkontakt (kg)*				**<0,001**
Mittelwert (SD)	126 (21,6)	153 (25,3)	133 (26,0)	
Median [Min, Max]	124 [74,0, 231]	150 [73,0, 280]	130 [73,0, 280]	
*BMI bei Erstkontakt*				**<0,001**
Mittelwert (SD)	45,7 (7,24)	47,3 (7,29)	46,2 (7,29)	
Median [Min, Max]	44,8 [26,2, 94,3]	46,4 [26,5, 71,4]	45,2 [26,2, 94,3]	
*Partnerschaft*				0,108
Ja	1165 (67,5 %)	429 (64,2 %)	1594 (66,6 %)	
Nein	543 (31,5 %)	234 (35,0 %)	777 (32,5 %)	
Unbekannt	17 (1,0 %)	5 (0,7 %)	22 (0,9 %)	
*Kinder*				**<0,001**
Mittelwert (SD)	1,42 (1,30)	1,05 (1,21)	1,31 (1,28)	
Median [Min, Max]	1,00 [0, 7,00]	1,00 [0, 6,00]	1,00 [0, 7,00]	
Unbekannt	31 (1,8 %)	12 (1,8 %)	43 (1,8 %)	
*Bildung*				**<0,001**
Hochschule	152 (8,8 %)	70 (10,5 %)	222 (9,3 %)	
Lehre, Fach‑/Meister‑/Technikerschule	749 (43,4 %)	376 (56,3 %)	1125 (47,0 %)	
Noch in Ausbildung	65 (3,8 %)	17 (2,5 %)	82 (3,4 %)	
Keine Angabe	388 (22,5 %)	103 (15,4 %)	491 (20,5 %)	
Andere^a^	334 (19,4 %)	82 (12,3 %)	416 (17,4 %)	
Unbekannt	37 (2,1 %)	20 (3,0 %)	57 (2,4 %)	

*BMI* Body-Mass-Index, *SD* Standardabweichung

^a^Andere: sonstige, oben nicht aufgeführte Bildungsart

Die Studienpopulation umfasste 1725 (72,1 %) weibliche und 668 (27,9 %) männliche Patient*innen (gesamt *n* = 2393). Männer waren mit einem Durchschnittsalter von 42,8 Jahren (± 12,0) beim Erstkontakt mit dem bariatrischen Zentrum signifikant älter als Frauen (41,3 Jahre [± 11,8], *p* = 0,007). Bei Erstkontakt betrug das durchschnittliche Gewicht bei Frauen 126 kg (± 21,6) und bei Männern 153 kg (± 25,3; *p* < 0,001). Der BMI beim ersten Besuch lag bei Frauen durchschnittlich bei 45,7 (± 7,24) kg/m^2^ und bei Männern bei 47,3 (± 7,29) kg/m^2^ (*p* < 0,001). Zum Zeitpunkt der Operation (also nach Durchlaufen eines 6‑monatigen, multimodalen, konservativen und nichtmedikamentösen Programms zur Gewichtsreduktion) betrug das durchschnittliche Gewicht der Frauen 127 kg (± 21,9) und das der Männer 158 kg (± 26,0; *p* < 0,001). Der BMI lag zu diesem Zeitpunkt bei Frauen bei durchschnittlich 46,2 (± 7,04) und bei Männern bei 48,3 (± 7,50; *p* < 0,001). Dies unterstreicht nochmals die weitgehende Wirkungslosigkeit verhaltensmodulierender Maßnahmen zur Gewichtsreduktion.

Insgesamt 67,5 % der Frauen und 64,2 % der Männer waren in einer Partnerschaft (*p* = 0,108). Die durchschnittliche Anzahl der Kinder lag bei Frauen bei 1,42 (± 1,30) und bei Männern bei 1,05 (± 1,21; *p* < 0,001). Der Bildungsgrad der männlichen Patienten, die sich zur Beratung bez. einer bariatrischen Intervention in unserem Zentrum vorstellten, war signifikant höher als der Bildungsgrad der weiblichen Patientinnen; so hatten 8,8 % der Frauen und 10,5 % der Männer einen Hochschulabschluss (*p* < 0,001).

### Adipositasassoziierte Begleiterkrankungen

Bei Erstkontakt litten 92,1 % der weiblichen und 91,3 % der männlichen Patienten an Begleiterkrankungen (*p* = 0,452). Typ-1-Diabetes war mit 0,8 % bei Frauen und 0,1 % bei Männern erwartungsgemäß selten (*p* = 0,131), während Typ-2-Diabetes (26,3 % vs. 17,3 %, *p* < 0,001), arterielle Hypertonie (59,1 % vs. 42,7 %, *p* < 0,001) und obstruktive Schlafapnoe (21,0 % vs. 20,4 %, *p* < 0,001) bei Männern signifikant häufiger vorkamen (Tab. 2).

**Tab. 2:** Begleiterkrankungen der Patient*innen bei Erstvorstellung

	Frauen (*n* = 1725)	Männern (*n* = 668)	Gesamt (*n* = 2393)	*p*-Wert
*Begleiterkrankungen*				0,452
Ja	1589 (92,1 %)	610 (91,3 %)	2199 (91,9 %)	
Nein	134 (7,8 %)	58 (8,7 %)	192 (8,0 %)	
Unbekannt	2 (0,1 %)	0 (0 %)	2 (0,1 %)	
*Diabetes mellitus Typ 1*				0,131
Ja	13 (0,8 %)	1 (0,1 %)	14 (0,6 %)	
Nein	1563 (90,6 %)	606 (90,7 %)	2169 (90,6 %)	
Unbekannt	149 (8,6 %)	61 (9,1 %)	210 (8,8 %)	
*Diabetes mellitus Typ 2*				**<0,001**
Ja	298 (17,3 %)	176 (26,3 %)	474 (19,8 %)	
Nein	1258 (72,9 %)	424 (63,5 %)	1682 (70,3 %)	
Unbekannt	169 (9,8 %)	68 (10,2 %)	237 (9,9 %)	
*Bluthochdruck*				**<0,001**
Ja	737 (42,7 %)	395 (59,1 %)	1132 (47,3 %)	
Nein	840 (48,7 %)	202 (30,2 %)	1042 (43,5 %)	
Unbekannt	148 (8,6 %)	71 (10,6 %)	219 (9,2 %)	
*Schlafapnoe*				**<0,001**
Ja	352 (20,4 %)	140 (21,0 %)	666 (27,8 %)	
Nein	1047 (60,7 %)	240 (35,9 %)	1287 (53,8 %)	
Unbekannt	326 (18,9 %)	288 (43,1 %)	440 (18,4 %)	
*Anzahl der Vorerkrankungen*				**<0,001**
0	567 (32,9 %)	95 (14,2 %)	662 (27,7 %)	
1	468 (27,2 %)	161 (24,1 %)	629 (26,3 %)	
2	229 (13,3 %)	199 (29,8 %)	428 (17,9 %)	
3	80 (4,6 %)	80 (12,0 %)	160 (6,7 %)	
Unbekannt	381 (22,1 %)	133 (19,9 %)	514 (21,5 %)	
*Dauermedikation*				0,187
Ja	1188 (68,9 %)	441 (66,0 %)	1629 (68,1 %)	
Nein	534 (31,0 %)	226 (33,8 %)	760 (31,8 %)	
Unbekannt	3 (0,2 %)	1 (0,1 %)	4 (0,2 %)	

Multimorbidität war bei Männern signifikant häufiger anzutreffen als bei Frauen. Während 58,1 % der Frauen an keiner oder nur einer adipositasassoziierten Begleiterkrankung litten, war dies nur bei 32,9 % der männlichen Patienten der Fall. Umgekehrt hatten 41,8 % der Männer und lediglich 17,9 % der Frauen zwei oder mehr Adipositasfolgeerkrankungen. Interessanterweise führte dieses Phänomen nicht zu Unterschieden bei der Häufigkeit einer Dauermedikation (68,9 % bei Frauen und 66,0 % bei Männern, *p* = 0,187).

### Perioperative Daten

Als hausinterner Standard empfehlen wir Patient*innen mit einer Indikation zur bariatrischen Intervention nach der aktuellen S3-Leitlinie „Chirurgie der Adipositas und metabolischer Erkrankungen“ und einem BMI <50 kg/m^2^ einen proximalen Roux-Y-Magenbypass („Roux-en‑Y gastric bypass“, RYGB). Die biliäre Schlingenlänge liegt standardmäßig bei 60 cm, die der alimentären Schlinge bei 150 cm. Gründe für ein Abweichen von diesem Standard können z. B. der Patient*innenwunsch, bekannte intraabdominelle Verwachsungen, die den Schlingenhochzug zum RYGB erschweren würden, oder bestimmte orale Medikationen sein. Bei einem BMI von ≥50 kg/m^2^ ist aufgrund des höheren Schwierigkeitsgrades von Operationen bei extremer Adipositas unser Standardverfahren die Sleeve-Gastrektomie (SG), ggf. gefolgt von einem weiteren Verfahren nach initialem Gewichtsverlust (z. B. sekundärer RYGB oder „single anastomosis duodeno-ileal bypass“ [SADI]). Andere Verfahren (z. B. der Einanastomosenbypass [„one anastomosis gastric bypass“, OAGB]) werden in unserem Zentrum als Ersteingriff nur in Ausnahmefällen durchgeführt. Aufgrund der Unterschiede in Operationstechnik und Komplikationsspektrum werden die Patient*innen im Folgenden nach Operationsverfahren getrennt analysiert. Die Verteilung der Operationstechniken über die Geschlechter findet sich in Tab. 3.

**Tab. 3:** Operationsmethoden

Operation	Frauen (*n* = 1725) (%)	Männer (*n* = 668) (%)	Gesamt (*n* = 2393) (%)	*p*-Wert
RYGB	614 (35,6)	127 (19,0)	741 (31,0)	**<0,001**
SG	319 (18,5)	155 (23,2)	474 (19,8)	
Keine Operation^a^	792 (45,9)	386 (57,8)	1178 (49,2)	

*RYGB* Roux-Y-Magenbypass, *SG* Sleeve-Gastrektomie

^a^Patient*innen ohne Operation haben sich entweder gegen eine Operation entschieden oder sich in einem anderen Zentrum operieren lassen

Bei 614 Frauen und 127 Männern (insgesamt *n* = 741) wurde ein RYGB durchgeführt (Tab. 4). Die durchschnittliche Operationsdauer lag bei Frauen bei 77,9 min (± 31,0) und bei Männern bei 88,7 min (± 33,8; *p* < 0,001). Nahezu alle Operationen wurden laparoskopisch durchgeführt (Frauen: 98,2 %; Männer: 96,9 %). Simultane Cholezystektomien wurden bei 1,1 % der Frauen und bei keinem Mann durchgeführt (*p* = 0,61). Simultane Adhäsiolysen fanden bei 0,7 % der Frauen und 2,4 % der Männer statt (*p* = 0,102). Intraoperative Komplikationen (z. B. Blutungen, Verletzungen anderer Organe) traten bei 0,7 % der Frauen und 0,8 % der Männer auf (*p* = 1). Nach der Clavien-Dindo-Klassifikation [6] erlitten 5,4 % der Frauen und 6,3 % der Männer leichtgradige Komplikationen (Grad ≤ 3a), während schwerwiegende Komplikationen (Grad $\geq$3b) extrem selten und in unserer Kohorte nur bei Frauen (2,1 %) auftraten (*p* = 0,222). Insgesamt zeigten sich bei den Komplikationen keine signifikanten Unterschiede zwischen den Geschlechtern. 87,7 % der Patient*innen erlitten überhaupt keine Komplikationen (Frauen: 87,3 %; Männer: 89,8 %; Tab. 4).

**Tab. 4:** Perioperative Daten – Roux-Y-Magenbypass

	Frauen (*n* = 614)	Männer (*n* = 127)	Gesamt (*n* = 741)	*p*-Wert
*Operationsdauer (min)*				**<0,001**
Mittelwert (SD)	77,9 (31,0)	88,7 (33,8)	79,8 (31,7)	
Median [Min, Max]	69,0 [28,0, 238]	84,0 [38,0, 271]	72,0 [28,0, 271]	
Unbekannt	5 (0,8 %)	1 (0,8 %)	6 (0,8 %)	
*Simultane Cholezystektomie*				0,61
Ja	7 (1,1 %)	0 (0 %)	7 (0,9 %)	
Nein	606 (98,7 %)	127 (100 %)	733 (98,9 %)	
Unbekannt	1 (0,2 %)	0 (0 %)	1 (0,1 %)	
*Simultane Adhäsiolyse*				0,102
Ja	4 (0,7 %)	3 (2,4 %)	7 (0,9 %)	
Nein	609 (99,2 %)	124 (97,6 %)	733 (98,9 %)	
Unbekannt	1 (0,2 %)	0 (0 %)	1 (0,1 %)	
*Intraoperative Komplikationen*				1
Ja	4 (0,7 %)	1 (0,8 %)	5 (0,7 %)	
Nein	609 (99,2 %)	126 (99,2 %)	735 (99,2 %)	
Unbekannt	1 (0,2 %)	0 (0 %)	1 (0,1 %)	
*Komplikationen (nach Clavien-Dindo)*				0,222
≥3b	13 (2,1 %)	0 (0 %)	13 (1,8 %)	
≤3a	33 (5,4 %)	8 (6,3 %)	41 (5,5 %)	
*Alle Clavien-Dindo-Grade*				0,802
Grad 1	10 (1,6 %)	2 (1,6 %)	12 (1,6 %)	
Grad 2	8 (1,3 %)	2 (1,6 %)	10 (1,3 %)	
Grad 3a	15 (2,4 %)	4 (3,1 %)	19 (2,6 %)	
Grad 3b	11 (1,8 %)	0 (0 %)	11 (1,5 %)	
Grad 4a	1 (0,2 %)	0 (0 %)	1 (0,1 %)	
Grad V	1 (0,2 %)	0 (0 %)	1 (0,1 %)	
Unbekannt	32 (5,2 %)	5 (3,9 %)	37 (5,0 %)	
Keine Komplikationen	536 (87,3 %)	114 (89,8 %)	650 (87,7 %)	

*SD* Standardabweichung

Bei 319 Frauen und 155 Männern (insgesamt *n* = 474) wurde eine SG durchgeführt (Tab. 5). Die durchschnittliche Operationsdauer lag bei Frauen bei 62,7 min (± 24,6) und bei Männern bei 70,5 min (± 25,0) (*p* < 0,001). Auch hier wurden nahezu alle Operationen laparoskopisch durchgeführt (Frauen: 99,1 %; Männer: 100 %). Simultane Cholezystektomien wurden bei 0,9 % der Frauen und bei keinem Mann durchgeführt (*p* = 0,554). Simultane Adhäsiolysen fanden bei 1,6 % der Frauen und 4,5 % der Männer statt (*p* = 0,066). Intraoperative Komplikationen (z. B. Blutungen, Verletzungen anderer Organe) traten bei 0,3 % der Frauen und 0,6 % der Männer auf (*p* = 0,548). Bei 4,7 % der Frauen und 3,9 % der Männer traten leichtgradige Komplikationen (Grad ≤ 3a) auf, schwerwiegende Komplikationen (Grad $\geq$3b) bei 1,6 % der Frauen und 4,5 % der Männer (*p* = 0,155). Insgesamt zeigten sich auch bei der Sleeve-Gastrektomie bezüglich der Komplikationsraten keine signifikanten Unterschiede zwischen den Geschlechtern (*p* = 0,458, Tab. 5).

**Tab. 5:** Perioperative Daten – Sleeve-Gastrektomie

	Frauen (*n* = 319)	Männer (*n* = 155)	Gesamt (*n* = 474)	*p*-Wert
*Operationsdauer (min)*				**<0,001**
Mittelwert (SD)	62,7 (24,6)	70,5 (25,0)	65,2 (25,0)	
Median [Min, Max]	59,0 [31,0, 193]	66,0 [35,0, 200]	60,0 [31,0, 200]	
Unbekannt	1 (0,3 %)	0 (0 %)	1 (0,2 %)	
*Simultane Cholezystektomie*				0,554
Ja	3 (0,9 %)	0 (0 %)	3 (0,6 %)	
Nein	316 (99,1 %)	155 (100 %)	471 (99,4 %)	
*Simultane Adhäsiolyse*				0,066
Ja	5 (1,6 %)	7 (4,5 %)	12 (2,5 %)	
Nein	314 (98,4 %)	148 (95,5 %)	462 (97,5 %)	
*Intraoperative Komplikationen*				0,548
Ja	1 (0,3 %)	1 (0,6 %)	2 (0,4 %)	
Nein	318 (99,7 %)	154 (99,4 %)	472 (99,6 %)	
*Komplikationen (nach Clavien-Dindo)*				0,155
≥3b	5 (1,6 %)	7 (4,5 %)	12 (2,5 %)	
≤3a	15 (4,7 %)	6 (3,9 %)	21 (4,4 %)	
*Alle Clavien-Dindo-Grade*				0,458
Grad 1	8 (2,5 %)	3 (1,9 %)	11 (2,3 %)	
Grad 2	3 (0,9 %)	1 (0,6 %)	4 (0,8 %)	
Grad 3a	4 (1,3 %)	2 (1,3 %)	6 (1,3 %)	
Grad 3b	5 (1,6 %)	7 (4,5 %)	12 (2,5 %)	
Unbekannt	24 (7,5 %)	15 (9,7 %)	39 (8,2 %)	
Keine Komplikationen	275 (86,3 %)	127 (81,9 %)	402 (84,8 %)	

*SD* Standardabweichung

Insbesondere bei Anastomoseninsuffizienzen bzw. Klammernahtinsuffizienzen konnte aufgrund der extremen Seltenheit dieser Komplikationen weder beim RYGB noch bei der SG ein signifikanter Unterschied zwischen den Geschlechtern gefunden werden (Daten nicht gezeigt).

### Postoperativer Verlauf

#### Gewicht

**Abb. 1:**
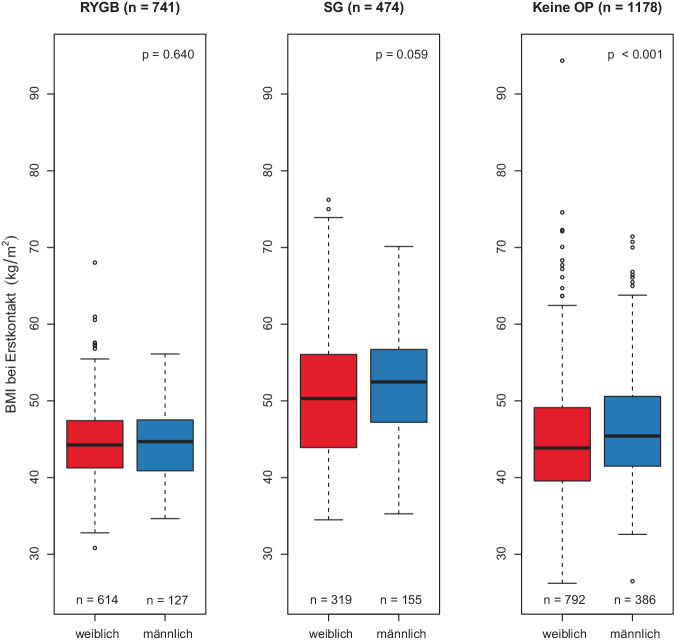
Body-Mass-Index (*BMI*) bei Erstkontakt, unterteilt nach zukünftigem Behandlungsverlauf. Der deutlich höhere BMI von Patient*innen, die schließlich eine Sleeve-Gastrektomie erhalten, erklärt sich durch die unterschiedliche Indikationsstellung (Patient*innen mit BMI >50 erhalten in unserem Zentrum beim Ersteingriff standardmäßig eine Sleeve-Gastrektomie). *OP* Operation, *RYGB* Roux-Y-Magenbypass, *SG* Sleeve-Gastrektomie

In der RYGB-Gruppe lag der BMI bei Erstkontakt bei 44,3 (± 4,73) kg/m^2^ (Frauen) bzw. 44,4 (± 4,46) kg/m^2^ (Männer; Abb. 1). Zum Zeitpunkt der Operation, also nach Durchlaufen eines 6‑monatigen konservativen Therapieversuchs, betrug der BMI im Mittel 43,8 (± 4,53) kg/m^2^ (Frauen) bzw. 44,1 (± 4,29) kg/m^2^ Männer. Im ersten Nachuntersuchungszeitraum (FU1) sank der BMI deutlich auf durchschnittlich 35,0 (± 4,65) kg/m^2^ bei Frauen, bei Männern auf 34,8 (± 4,23) kg/m^2^. Dieser Trend setzte sich im zweiten Nachuntersuchungszeitraum (FU2) fort (Frauen: 30,0 [± 4,61], Männer: 31,4 [± 4,37]). Im zweiten postoperativen Jahr (zwischen FU2 und FU3) blieb der BMI bei beiden Geschlechtern nach RYGB relativ stabil. Insgesamt verloren Frauen in den ersten 24 Monaten nach RYGB durchschnittlich 14,2 und Männer 12,9 BMI-Punkte.

**Abb. 2:**
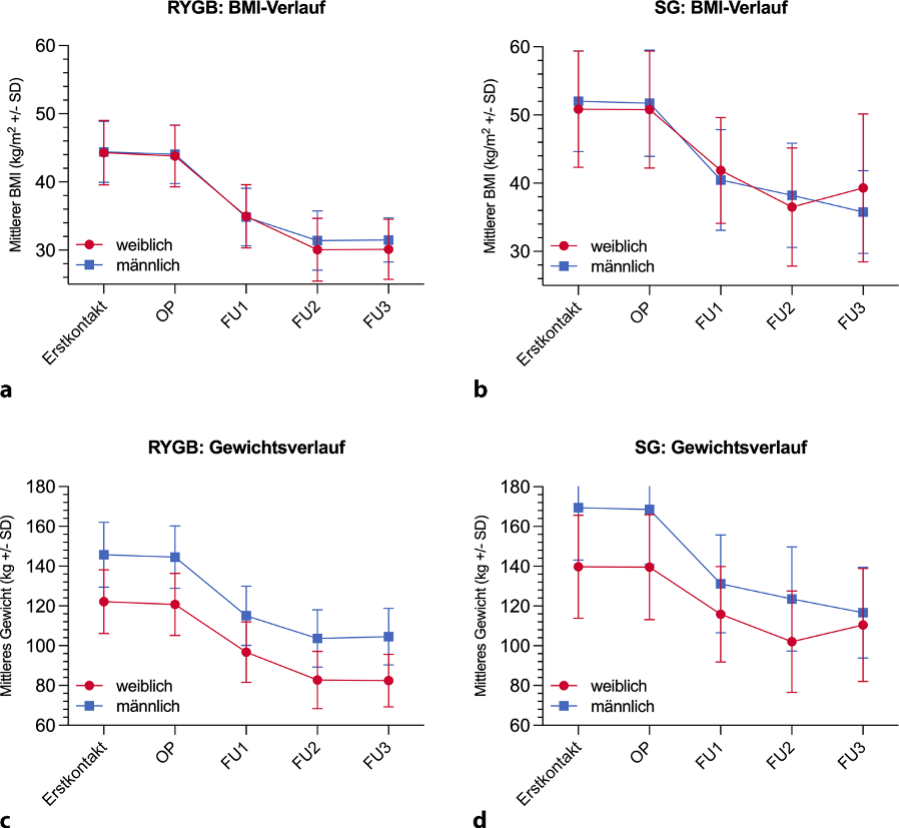
Gewichtsverlauf nach bariatrischer Operation. BMI nach RYGB (**a**) oder SG (**b**). Absolutes Gewicht nach RYGB (**c**) oder SG (**d**). *BMI* Body-Mass-Index, *FU* Follow-up, *OP* Operation, *RYGB* Roux‑Y Magenbypass, *SD* Standardabweichung, *SG* Sleeve-Gastrektomie

In der SG-Gruppe lag der BMI bei Erstkontakt aufgrund der anderen Indikationsstellung (s. oben) bei 50,9 (± 8,52; Frauen) bzw. 52,0 (± 7,40; Männer) und damit deutlich höher. Zum Zeitpunkt der Operation betrug der BMI im Mittel 50,8 (± 8,58) bei Frauen und 51,7 (± 7,80) bei Männern (Abb. 1). Im ersten Nachuntersuchungszeitraum (FU1) sank der BMI signifikant auf durchschnittlich 41,9 (± 7,78) bei Frauen und auf 40,5 (± 7,38) bei Männern. Dieser Trend setzte sich im weiteren Verlauf des ersten postoperativen Jahres bis FU2 fort (Frauen: 36,5 [± 8,65], Männer: 38,2 [± 7,66]). Im zweiten postoperativen Jahr zeigte sich interessanterweise eine Divergenz: Während bei Frauen der BMI wieder zunahm (bis 39,3 ± 10,8), nahmen Männer im zweiten Jahr weiter ab auf 35,8 (± 6,09). Insgesamt verloren Frauen in den ersten 24 Monaten nach SG durchschnittlich 12,6 und Männer 16,2 BMI-Punkte. Aufgrund der zum letzten Nachsorgezeitpunkt wenigen verfügbaren Datensätze konnte hier kein statistisch signifikanter Unterschied gezeigt werden. Die Daten zum postoperativen BMI- und Gewichtsverlauf sind in den Tabellen S5 und S6 sowie in Abb. 2 dargestellt.

**Abb. 3:**
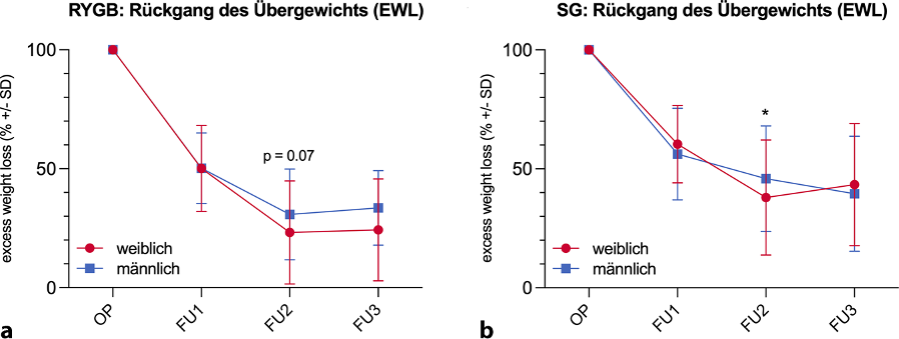
Abnahme des Übergewichts („excess weight loss“, *EWL*) im postoperativen Verlauf nach RYGB (**a**) und SG (**b**). *FU* Follow-up, *OP* Operation,* RYGB* Roux-Y-Magenbypass, *SD* Standardabweichung, *SG* Sleeve-Gastrektomie. *p < 0,05

Zur besseren Vergleichbarkeit der Daten zwischen den Geschlechtern wurde die prozentuale Veränderung des Übergewichts („excess weight loss“, EWL) berechnet (Abb. 3). Hier zeigte sich, dass Frauen 12 Monate nach RYGB mit 76,8 % (± 21,7) einen in der Tendenz (*p* = 0,07) höheren Prozentsatz ihres Übergewichts verloren hatten als Männer (69,2 % ± 19,2), was sich auch 24 Monate postoperativ noch nachweisen ließ. Beim SG zeigte sich 12 Monate nach der Operation sogar ein statistisch signifikant höherer Rückgang des Übergewichts bei Frauen im Vergleich zu Männern (62,1 % ± 24,2 vs. 54,2 % ± 22,2, *p* = 0,04). Insgesamt war der Rückgang des Übergewichts beim RYGB mit 75,4 % deutlich ausgeprägter als beim SG (60,0 %).

#### Postoperative Entwicklung adipositasassoziierter Erkrankungen

Die Prävalenz adipositasassoziierter Erkrankungen im Verlauf der bariatrischen Behandlungen ist in den Tabellen S2–S4 im Onlinezusatzmaterial dargestellt. Zusammengefasst konnten keine signifikanten Unterschiede zwischen den Geschlechtern detektiert werden.

## Diskussion

Die Adipositas ist eine chronische Erkrankung, die im Verlauf adipositasassoziierte Folgeerkrankungen nach sich zieht und so eine erhebliche Morbidität und Mortalität verursacht. Aufgrund der hohen und weiter steigenden Prävalenz stellt sie ein erhebliches epidemiologisches und gesundheitspolitisches Problem dar. Adipositaschirurgische Eingriffe sind eine effektive Behandlungsmethode, die sowohl Morbidität als auch Mortalität langfristig deutlich verbessern können [5, 9, 14, 15].

Bei der Manifestation der Adipositas zeigen sich signifikante geschlechtsspezifische Unterschiede: Obwohl Frauen generell einen höheren Körperfettanteil haben, ist die Prävalenz der Adipositas (definiert als ein BMI ≥30 kg/m^2^) in Deutschland bei Männern höher. Chirurgisch relevant ist hier insbesondere die unterschiedliche Fettverteilung: Während Frauen in der Tendenz eher zu einer peripheren und subkutanen Adipositas neigen, konzentriert sich die Adipositas beim männlichen Geschlecht eher auf das viszerale Fett, während die Bauchdecke auch bei extrem adipösen Patient*innen vergleichsweise dünn bleibt. Dieses intraabdominelle Fettgewebe erschwert abdominelle Operationen deutlich.

Während in den USA die Prävalenz der Adipositas bei Frauen höher ist als bei Männern [7], sind in Deutschland mit 24,2 % vs. 20,4 % Männer häufiger adipös [18]. Die hier vorgestellten Daten zeigen erstmals die geschlechtsspezifischen Unterschiede in der bariatrischen Versorgung in Deutschland. Dabei decken sich die epidemiologischen Eckpunkte der hier vorgestellten Patient*innenkohorte bezüglich der Geschlechterverteilung, des Alters, der Komorbiditäten und des initialen BMI weitgehend mit den aktuell berichteten internationalen Daten der IFSO [4, 10]. Lediglich die primäre Operationstechnik unterscheidet sich, da in unserem Zentrum der RYGB aufgrund seiner metabolischen Effekte und des im Mittel höheren Gewichtsverlusts bevorzugt wird, während national und international die SG als technisch einfacherer Eingriff häufiger durchgeführt wird.

Analog zu den internationalen Daten zeigte sich in unserer Kohorte, dass trotz der höheren Inzidenz der Adipositas bei Männern hierzulande die Frauen in der bariatrischen Versorgung stark überrepräsentiert sind. Die Gründe hierfür sind sicher vielfältig. So unterliegen Frauen mutmaßlich einem größeren sozialen Druck, ästhetischen Normen zu folgen, und die Vorstellung des idealen weiblichen Körpers ist immer noch geprägt durch Models, die eher sogar als untergewichtig gelten können. Dieses omnipräsente Schönheitsideal und die Stigmatisierung des normal- bis übergewichtigen weiblichen Körpers („body shaming“) führt dazu, dass adipöse Frauen einem höheren Leidensdruck ausgesetzt sind als Männer mit gleichem BMI, was zu einer größeren Therapiemotivation führen könnte. Darüber hinaus haben Frauen im Allgemeinen ein stärkeres Bewusstsein, was Gesundheitsthemen und -vorsorge angeht. Dazu passend haben Männer, die sich in unserem bariatrischen Zentrum vorstellten, einen signifikant höheren Bildungsgrad als Frauen in bariatrischer Behandlung. Es scheint wahrscheinlich, dass Männer aufgrund des geringeren ästhetischen Leidensdrucks erst ab einem bestimmten Bildungsgrad ausreichend über die Folgeerkrankungen der Adipositas informiert und so für eine bariatrische Intervention motiviert sind. Da jedoch gerade das männliche Fettverteilungsmuster metabolische Folgeerkrankungen begünstigt, ist offensichtlich, dass insbesondere Männer in Deutschland noch nicht ausreichend erreicht und für eine Behandlung ihrer Adipositas motiviert werden können.

Männer werden in Deutschland nicht ausreichend für eine Behandlung ihrer Adipositas motiviert

Ein weiterer Unterschied zwischen den Geschlechtern ist die deutlich höhere Morbidität der männlichen Adipositaspatienten bei Erstvorstellung. Dies lässt sich ebenfalls durch die geringere Motivation von Männern zur bariatrischen Behandlung und damit späterer Erstvorstellung erklären, außerdem entwickeln Männer aufgrund der viszeralen Fettverteilung und der metabolisch ungünstigen Folgen viszeraler Adipositas früher und häufiger Adipositasfolgeerkrankungen. Das geringere Bewusstsein für Gesundheitsfragen unter Männern scheint auch durch die (statistisch nicht signifikante) geringere Prävalenz einer Dauermedikation bei gleichzeitig deutlich höherer Morbidität der männlichen Patienten bei Erstvorstellung bestätigt zu werden.

Perioperativ ergaben sich bei der geschlechtsspezifischen Analyse unserer Kohorte keine Überraschungen: So unterzogen sich mehr als dreimal mehr Frauen als Männer einem bariatrischen Eingriff, bei Frauen wurden aufgrund des im Mittel niedrigeren BMI signifikant mehr RYGBs als SGs durchgeführt, während bei Männern die SG der häufigere Eingriff war. Aufgrund der unterschiedlichen Operationstechnik wurden die perioperativen Parameter separat nach Eingriffsart analysiert, auch hier zeigte sich jedoch wenig überraschend, dass beide Operationstechniken bei Männern aufgrund der mehrheitlich intraabdominellen Adipositas und daraus resultierender technischer Erschwernisse signifikant länger dauern als bei Frauen. Dies übersetzte sich jedoch nicht in eine höhere Komplikationsrate bei Männern.

Auch der postoperative Verlauf der hier vorgestellten Kohorte entspricht weitgehend anderen, internationalen Kohorten. Der Nadir des Gewichts ist um den 12. postoperativen Monat erreicht [13, 16], gefolgt von einem diskreten Wiederanstieg. Im Gegensatz zu anderen bariatrischen Kohorten konnte in unserer Studienpopulation kein signifikanter Rückgang adipositasassoziierter Erkrankungen gezeigt werden; dies ist jedoch am ehesten auf eine lückenhafte Datenerhebung als auf tatsächliche Effekte zurückzuführen. Der prozentuale Rückgang des Übergewichts (EWL) bewegte sich ebenfalls im erwarteten Bereich.

Der Nadir des Gewichts ist um den 12. postoperativen Monat erreicht

Ein derzeit hochaktuelles Thema sind GLP-1-Agonisten wie z. B. Semaglutid, die als medikamentöse Behandlungsform das Potenzial haben, dauerhaft eine große Rolle in der Therapie der Adipositas einzunehmen. Aufgrund der fehlenden Langzeitdaten insbesondere zur Sicherheit dieser dauerhaften Medikation und ihrer im Vergleich zur MBS geringeren Effektivität wird die MBS allerdings auf absehbare Zeit ihren Stellenwert behalten. Während bei Patient*innen mit leichtem bis moderatem Übergewicht der Einsatz von GLP-1-Agonisten eine ausreichende Gewichtsreduktion und Verbesserung der Stoffwechselparameter bewirken kann, sehen wir die Rolle von GLP-1-Agonisten eher bei Patient*innen mit hochgradiger Adipositas in der Neoadjuvanz vor einem bariatrischen Eingriff im Sinne eines multimodalen Konzepts. Auch die medikamentöse Adipositastherapie ist beim weiblichen Geschlecht stark überrepräsentiert [1, 12], darüber hinaus liegen derzeit keine geschlechtsspezifische Daten zu GLP-1-Agonisten in der Adipositastherapie vor.

Insgesamt zeigen die hier vorgelegten Daten, dass Männer trotz einer höheren Prävalenz der Adipositas in Deutschland noch nicht ausreichend angesprochen und für eine Therapie ihres Übergewichts gewonnen werden können. Diese Versorgungslücke gilt es zu schließen, um die bekannten negativen Auswirkungen der chronischen Adipositas auf die Morbidität und Mortalität der Bevölkerung und das Gesundheitssystem insgesamt abzumildern.

## Fazit für die Praxis

- Männer haben in unserem Kollektiv signifikant höhere Body-Mass-Index-Werte und mehr adipositasassoziierte Erkrankungen als Frauen.
- Die metabolisch/bariatrische Chirurgie (MBS) führt bei beiden Geschlechtern zu einem vergleichbaren und signifikanten Rückgang des Übergewichts.
- Aktuell werden Männer trotz der höheren Prävalenz der Adipositas noch nicht ausreichend einer spezifischen Therapie der Adipositas zugeführt.
- Zukünftig sollte ein Fokus der medizinischen Versorgung sein, die Versorgungslücke vor allem bei Männern zu schließen, um die bekannten negativen Auswirkungen der chronischen Adipositas auf die Morbidität und Mortalität der Bevölkerung und das Gesundheitssystem zu reduzieren.

### Supplementary Information

Tabelle S1. Anzahl der durchgeführten Untersuchungen an den jeweiligen Nachsorgeterminen, Tabelle S2. Prävalenz der Hypertonie zu verschiedenen Nachsorgezeitpunkten, Tabelle S3. Prävalenz des Schlafapnoesyndroms zu verschiedenen Nachsorgezeitpunkten, Tabelle S4. Prävalenz von Diabetes mellitus Typ 2 zu verschiedenen Nachsorgezeitpunkten, Tabelle S5: BMI-Verlauf
