# Supplementary material for: Gender-specific differences in bariatric surgery: epidemiology, treatment and results
Source: Chirurgie (Heidelb). 2024 Aug 8;95(9):721–9. [Article in German] doi: 10.1007/s00104-024-02149-z (PMC11333528; doi:10.1007/s00104-024-02149-z)
Supplement: Supplementary file 1 — Tabelle S1. Anzahl der durchgeführten Untersuchungen an den jeweiligen Nachsorgeterminen, Tabelle S2. Prävalenz der Hypertonie zu verschiedenen Nachsorgezeitpunkten, Tabelle S3. Prävalenz des Schlafapnoesyndroms zu verschiedenen Nachsorgezeitpunkten, Tabelle S4. Prävalenz von Diabetes mellitus Typ 2 zu verschiedenen Nachsorgezeitpunkten, Tabelle S5: BMI-Verlauf [file 104_2024_2149_MOESM1_ESM.pdf]

Tabelle S1. Anzahl der durchgeführten Untersuchungen an den jeweiligen Nachsorgeterminen.

	weiblich		männlich		gesamt	
	RYGB (n=1855)	SG (n=964)	RYGB (n=387)	SG (n=444)	RYGB (n=2242)	SG (n=1408)
**Zeitpunkt**						
Erstkontakt	614 (33.1%)	319 (33.1%)	127 (32.8%)	155 (34.9%)	741 (33.1%)	474 (33.7%)
OP	612 (33.0%)	318 (33.0%)	127 (32.8%)	155 (34.9%)	739 (33.0%)	473 (33.6%)
FU1 (3 Mo.)	336 (18.1%)	180 (18.7%)	76 (19.6%)	84 (18.9%)	412 (18.4%)	264 (18.8%)
FU2 (12 Mo.)	212 (11.4%)	112 (11.6%)	46 (11.9%)	40 (9.0%)	258 (11.5%)	152 (10.8%)
FU3 (24 Mo.)	71 (3.8%)	33 (3.4%)	11 (2.8%)	9 (2.0%)	82 (3.7%)	42 (3.0%)
FU4 (36 Mo.)	10 (0.5%)	2 (0.2%)	0 (0%)	1 (0.2%)	10 (0.4%)	3 (0.2%)

Tabelle S2. Prävalenz der Hypertonie zu verschiedenen Nachsorgezeitpunkten.

	Erstkontakt	FU1	FU2	FU3	Gesamt
**Hypertonie: Frauen / RYGB**					
Ja	285 (49.7%)	160 (54.2%)	89 (51.7%)	26 (53.1%)	560 (51.4%)
Nein	289 (50.3%)	135 (45.8%)	83 (48.3%)	23 (46.9%)	530 (48.6%)
unbekannt	40	319	442	565	1366
**Hypertonie: Männer / RYGB**					
Ja	82 (74.5%)	41 (58.6%)	25 (61.0%)	6 (54.5%)	154 (66.4%)
Nein	28 (25.5%)	29 (41.4%)	16 (39.0%)	5 (45.5%)	78 (33.6%)
unbekannt	17	57	86	116	276
**Hypertonie: Frauen / SG**					
Ja	138 (47.1%)	79 (48.8%)	44 (48.4%)	14 (56.0%)	275 (48.2%)
Nein	155 (52.9%)	83 (51.2%)	47 (51.6%)	11 (44.0%)	296 (51.8%)
unbekannt	26	157	228	294	705
**Hypertonie: Männer / SG**					
Ja	82 (59.0%)	45 (60.8%)	21 (61.8%)	4 (80.0%)	152 (60.3%)
Nein	57 (41.0%)	29 (39.2%)	13 (38.2%)	1 (20.0%)	100 (39.7%)
unbekannt	16	81	121	150	368

Tabelle S3. Prävalenz des Schlafapnoesyndroms zu verschiedenen Nachsorgezeitpunkten.

	Erstkontakt	FU1	FU2	FU3	Gesamt
**Schlafapnoe: Frauen / RYGB**					
Ja	126 (24.8%)	143 (48.5%)	71 (41.3%)	26 (53.1%)	366 (35.7%)
Nein	383 (75.2%)	152 (51.5%)	101 (58.7%)	23 (46.9%)	659 (64.3%)
unbekannt	105	319	442	565	1431
**Schlafapnoe: Männer / RYGB**					
Ja	63 (60.6%)	55 (78.6%)	28 (68.3%)	8 (72.7%)	154 (68.1%)
Nein	41 (39.4%)	15 (21.4%)	13 (31.7%)	3 (27.3%)	72 (31.9%)
unbekannt	23	57	86	116	282
**Schlafapnoe: Frauen / SG**					
Ja	82 (31.4%)	77 (47.5%)	36 (39.6%)	13 (52.0%)	208 (38.6%)
Nein	179 (68.6%)	85 (52.5%)	55 (60.4%)	12 (48.0%)	331 (61.4%)
unbekannt	58	157	228	294	737
**Schlafapnoe: Männer / SG**					
Ja	72 (55.8%)	51 (68.9%)	23 (67.6%)	3 (60.0%)	149 (61.6%)
Nein	57 (44.2%)	23 (31.1%)	11 (32.4%)	2 (40.0%)	93 (38.4%)
unbekannt	26	81	121	150	378

Tabelle S4. Prävalenz von Diabetes mellitus Typ 2 zu verschiedenen Nachsorgezeitpunkten.

	**Erstkontakt**	**FU1**	**FU2**	**FU3**	**Gesamt**
**DM2: Frauen / RYGB**					
Ja	96 (17.2%)	67 (22.7%)	36 (20.9%)	14 (28.6%)	213 (19.9%)
Nein	461 (82.8%)	228 (77.3%)	136 (79.1%)	35 (71.4%)	860 (80.1%)
unbekannt	57	319	442	565	1383
**DM2: Männer / RYGB**					
Ja	42 (37.8%)	28 (40.0%)	15 (36.6%)	5 (45.5%)	90 (38.6%)
Nein	69 (62.2%)	42 (60.0%)	26 (63.4%)	6 (54.5%)	143 (61.4%)
unbekannt	16	57	86	116	275
**DM2: Frauen / SG**					
Ja	59 (20.5%)	41 (25.3%)	19 (20.9%)	8 (32.0%)	127 (22.4%)
Nein	229 (79.5%)	121 (74.7%)	72 (79.1%)	17 (68.0%)	439 (77.6%)
unbekannt	31	157	228	294	710
**DM2: Männer / SG**					
Ja	25 (17.9%)	18 (24.3%)	9 (26.5%)	2 (40.0%)	54 (21.3%)
Nein	115 (82.1%)	56 (75.7%)	25 (73.5%)	3 (60.0%)	199 (78.7%)
unbekannt	15	81	121	150	367

Tabelle S5: BMI-Verlauf

	Erstkontakt	OP	FU1	FU2	FU3
**BMI zu verschiedenen Zeitpunkten (Frauen - RYGB)**	n=614	n=612	n=336	n=212	n=71
Mittelwert (SD)	44.3 (4.73)	43.8 (4.53)	35.0 (4.65)	30.0 (4.61)	30.1 (4.42)
Median [Min, Max]	44.3 [30.8, 68.0]	43.7 [31.1, 62.5]	35.0 [23.0, 48.7]	29.8 [19.7, 43.3]	30.1 [22.0, 41.9]
**BMI zu verschiedenen Zeitpunkten (Männer - RYGB)**	n=127	n=127	n=76	n=46	n=11
Mittelwert (SD)	44.4 (4.46)	44.1 (4.29)	34.8 (4.23)	31.4 (4.37)	31.5 (3.24)
Median [Min, Max]	44.7 [34.7, 56.1]	43.9 [34.7, 55.4]	34.3 [23.5, 45.0]	31.2 [19.5, 41.5]	31.1 [25.7, 36.4]
**BMI zu verschiedenen Zeitpunkten (Frauen - SG)**	n=319	n=318	n=180	n=112	n=33
Mittelwert (SD)	50.9 (8.53)	50.8 (8.58)	41.9 (7.78)	36.5 (8.65)	39.3 (10.8)
Median [Min, Max]	50.3 [34.5, 76.2]	50.7 [32.1, 79.5]	41.8 [25.3, 62.0]	36.2 [22.2, 67.0]	37.8 [23.7, 67.0]
**BMI zu verschiedenen Zeitpunkten (Männer - SG)**	n=155	n=155	n=84	n=40	n=9
Mittelwert (SD)	52.0 (7.40)	51.7 (7.80)	40.5 (7.38)	38.2 (7.66)	35.8 (6.09)
Median [Min, Max]	52.5 [35.3, 70.1]	52.1 [36.2, 73.3]	40.9 [22.7, 54.2]	37.9 [22.2, 53.1]	35.0 [23.5, 45.4]

Tabelle S6: Gewichtsverläufe

	Erstkontakt	OP	FU1	FU2	FU3
**Gewicht zu verschiedenen Zeitpunkten (Frauen - RYGB)**	n=614	n=612	n=336	n=212	n=71
Mittelwert (SD)	122 (16.0)	121 (15.6)	96.8 (15.3)	82.7 (14.4)	82.5 (13.2)
Median [Min, Max]	123 [76.0, 175]	120 [77.0, 170]	96.5 [61.0, 152]	83.0 [53.0, 130]	84.0 [59.0, 119]
**Gewicht zu verschiedenen Zeitpunkten (Männer - RYGB)**	n=127	n=127	n=76	n=46	n=11
Mittelwert (SD)	146 (16.3)	145 (15.8)	115 (14.9)	104 (14.5)	105 (14.2)
Median [Min, Max]	147 [99.0, 190]	144 [99.0, 182]	115 [78.0, 146]	104 [69.0, 133]	105 [84.0, 125]
**Gewicht zu verschiedenen Zeitpunkten (Frauen - SG)**	n=319	n=318	n=180	n=112	n=33
Mittelwert (SD)	140 (25.9)	140 (26.4)	116 (24.1)	102 (25.5)	110 (28.4)
Median [Min, Max]	136 [86.0, 229]	136 [78.0, 217]	114 [63.0, 176]	98.5 [54.0, 170]	102 [63.0, 175]
**Gewicht zu verschiedenen Zeitpunkten (Männer - SG)**	n=155	n=155	n=84	n=40	n=9
Mittelwert (SD)	169 (26.3)	169 (27.7)	131 (24.6)	124 (26.2)	117 (22.8)
Median [Min, Max]	170 [113, 240]	167 [111, 245]	132 [75.0, 186]	124 [68.0, 190]	114 [72.0, 157]
